# Efficient data acquisition and reconstruction for air-coupled ultrasonic robotic NDE

**DOI:** 10.1038/s41598-024-60393-z

**Published:** 2024-06-18

**Authors:** Ciaron Hamilton, Oleksii Karpenko, Lalita Udpa, Mahmoodul Haq, Yiming Deng

**Affiliations:** 1 Nondestructive Evaluation Laboratory (NDEL), Composite Vehicle Research Center (CVRC), Michigan State University College of Electrical and Computer Engineering, East Lansing, MI 48824-1226 USA; 2 Composite Vehicle Research Center (CVRC), Michigan State University College of Civil and Environmental Engineering, East Lansing, MI 48824-1226 USA

**Keywords:** Civil engineering, Electrical and electronic engineering, Mechanical engineering, Structural materials

## Abstract

Non-destructive evaluation of complex parts using surface scanning techniques, such as ultrasonic testing and eddy current testing, requires complex manipulation of such sensors to ensure quantitative results. A robotic arm may function as a complex manipulator for surface scanning, controlling the position and tilt between the probe and specimen’s surface. To ensure accuracy in probe manipulation, accurate geometric information of the specimen is required. This article explores a methodology that uses structured light for physical-to-virtual reconstruction, providing submillimeter scale and accurate surface geometries. Reconstruction aids in path planning through a novel ray-triangle intersection array algorithm, establishing movements for the NDE probe to orient itself on the specimen at a constant probe to specimen surface distance, or lift-off. The proposed technique is demonstrated and validated through experimental air-coupled ultrasonic inspection of automotive CFRP composite samples with simulated flaws such as interlaminar delamination. The proposed method employs guided waves and a pitch-catch configuration of air-coupled ultrasonic probes, enabling single-side access scans. A Fanuc 100ib robot arm was used to manipulate the ultrasonic probes along a sample reconstructed with a CR-Scan 01 structured light sensor. The probes were excited at 200khz from a SonoAir system, while also recovering defect vs background information synchronized with the probe’s orientation. Additionally, a framework for potential automation is proposed, with further details to be explored in future works.

## Introduction

Autonomous scanning and data acquisition play a pivotal role in the progression of NDE 4.0, bridging principles between NDE and industry 4.0 techniques through industrial internet of things (IIOT) and cyber-physical systems (CPS) implementations. One key advantage is that advanced digital NDE 3.0 inspection techniques, such as ultrasonic, eddy current, optical and X-ray, will be re-configured and employed in NDE 4.0, and facilitate enhanced detection of defects by navigating the probe along accurate paths or at more strategic sensing locations over the object under inspection. NDE 4.0 technology should also consider the complexity of damages which come in many different forms, specifically with sub-surface defects being challenging to detect.

NDE 4.0, with robotic systems, has valuable applications in industrial inspections of complex non-flat geometries. Hydro turbine blades exemplify the need for NDE examination to prevent critical failures, downtime, and risks to personnel^1–3^. Similarly, aircraft components with diverse geometries, prone to fatigue and stress-corrosion cracking, benefit from NDE inspection to avoid component failures that could lead to potential accidents^4,5^. In the energy sector, robotic NDE is crucial for assessing the health of power plant vessels. Robotic actuation devices, such as wall-climbing robots equipped with eddy current probes, play a critical role in inspecting non-compliant workspaces, including power plant boiler plates, liquid storage tanks, pipelines, and ship hulls^6–9^.

A common approach for planning scan paths in robotic NDE 4.0 on complex surfaces involves utilizing a pre-defined CAD model for path planning, often incorporating b-spline methods alongside the specific geometric definitions provided by CAD models^10^. However, validating the exact geometric complexity, particularly when complex pieces may deform over time compared to an expected virtual model, introduces discrepancies in probe pathing. Furthermore, a CAD model remains in a digital space until aligned with the desired orientation for scanning to the robot’s base frame. Ensuring correct alignment in practice can be challenging and time-consuming, especially in real-time operational environments where components may undergo slight repositioning during testing as in-line and in-service evaluations are vital to NDE 4.0.

**Figure 1:**
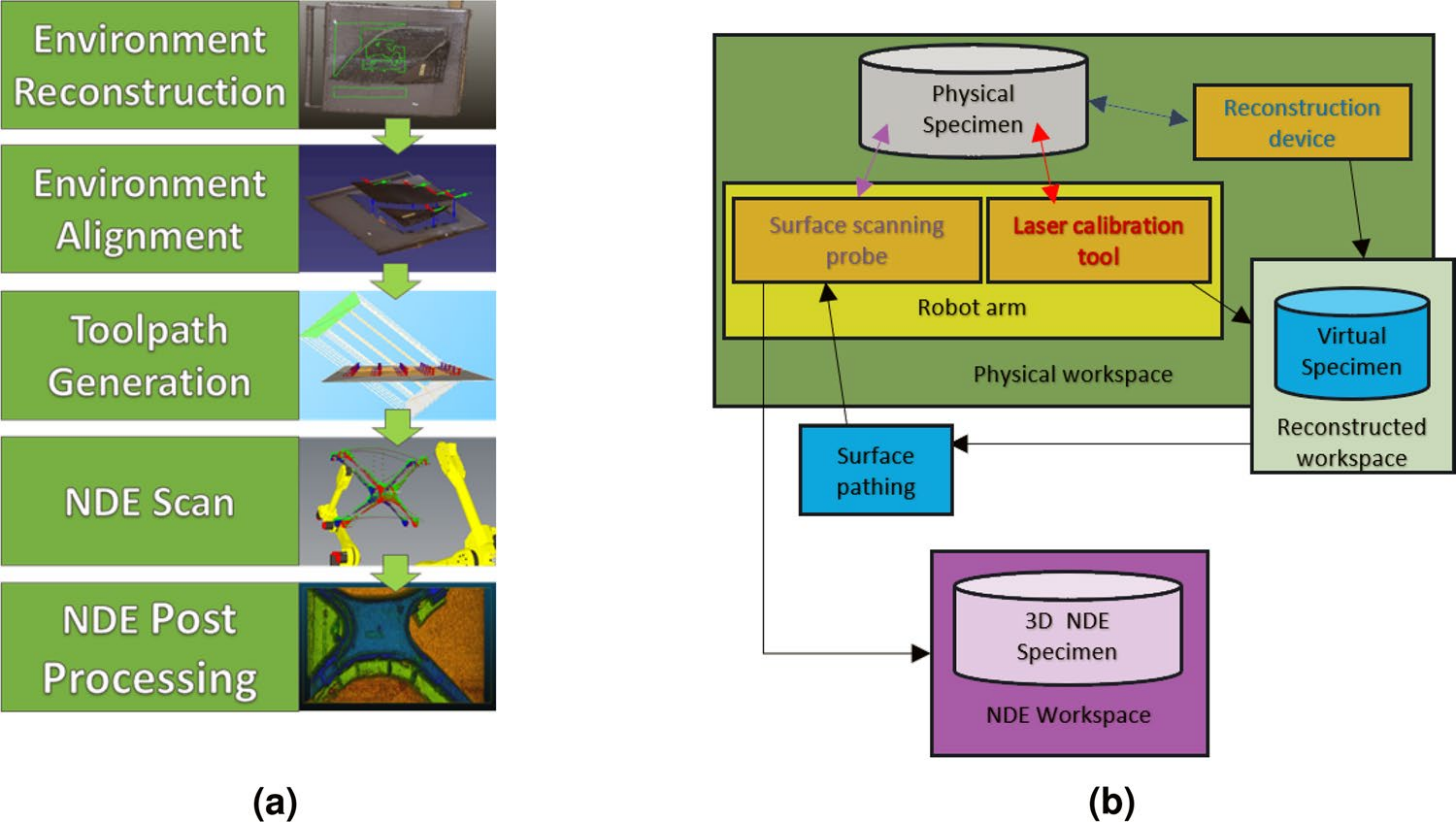
Framework and system layout. (**a**) Proposed 6-DOF NDE 4.0 framework, showing reconstruction, alignment, generation, scanning, and post processing. (**b**) System layout showing the robot, sensors, and flow of data.

To overcome the limitations associated with predefined CAD models, we propose a framework employing surface reconstruction for NDE inspection with robotic arms. This approach offers insights into the scanning environment during the component’s examination, eliminating previously mentioned disparities. The reconstructed environment serves a dual purpose, allowing scrutiny of background components for advanced NDE path planning and collision avoidance. The basic outline of the framework is shown in Fig. 1. Reconstruction will obtain a surface geometric profile. Registration aligns this geometric information to the robot’s workspace. To create the movements for the probe, toolpath generation employs a novel approach, using a ray-triangle intersection array algorithm to parse movements on the surface geometry. The physical movements are then generated and sent to the robot, which will synchronize the probe’s information with its orientation.

### Surface reconstruction for autonomous robotic NDE

Surface reconstruction may be used to autonomously obtain the surface details of an “inspection space,” including the geometry of the specimen which the robot may reach, and background components to avoid collision with. This “inspection space” can be considered as a 3D virtual environment, which are meshes or CADs containing important components for a robot to consider. Specifically, surface information of the specimen may be used to parse toolpaths to orient a probe, including its position and tilt. For reconstructed meshes, a device using methods such as structured light or blue light can be used to obtain a sub-millimeter accurate virtual model of physical objects. Meshes obtained this way require alignment to the robot’s base frame, else the frame will be aligned towards the devices local frame.

Ensuring higher accuracy in reconstruction and alignment is crucial for precise sensor positioning in NDE scanning and sensory data collection, particularly in techniques like air-coupled UT or eddy current scanning with low lift-off distances requirement for better energy coupling. The accuracy between virtual and physical geometries is beneficial for both complex and non-complex components, such as flat coupon samples. Inconsistent or large lift-off issues can lead to misleading or poor quantitative information in the output scan. Lift-off issues may be countered by advanced techniques such as with deep learning^11^ or transfer learning^12^ for different NDE surface scanning techniques. There is still, however, risk that no valuable information is picked up from poor positioning. Robotic systems, prone to errors, especially in stability, may require thorough examination of the impact of these errors on probe accuracy. Errors may occur due to imperfections in mastering or mechanical issues, including errors in the probe’s movement trajectory.

### State-of-the-art autonomous scans and surface reconstruction

Solutions addressing the inverse kinematic problem, achieving 6-DOF transformations for sensors on robot arm platforms, have been developed^13^. Dual ultrasonic testing (UT) robotic arm systems have demonstrated applications in scanning cylindrical composite objects using water-jet transmission^14^, as well as quality control of helicopter components with carbon-fiber reinforced polymer (CFRP) laminates^15,16^. Challenges arise in managing the complexity of helicopter components in such approaches. Synchronization of twin robot systems^17^ and the development of inspection and repair robot crawlers for power plant boiler inspections^6^ showcase progress in mobile platform systems. An in-line inspection system for cylindrical pipes, integrating endoscopic laser profiling and machine learning, addresses deformations within pipes^9^. Various imaging and reconstruction solutions include hyperspectral cameras for CFRP panel imaging using robot arms^18^, x-ray computed tomography for reference-free 3D scans to localize damages^19,20^, and the use of Structure from Motion techniques in robot arm environments^21^. Time-of-flight cameras find applications in environment reconstruction for both mobile robot platforms and robot arms^22^. Stereo cameras, explored for NDE and welding applications^23,24^, offer portable and fast computation, particularly when compared to methods like structured light. A crawler robot for NDE was designed for plate inspection using stereovision^25^. Thermography, suitable for inspecting complex parts such as wind turbines^26^, has been examined in conjunction with robotics^27^. An advanced state-of-the-art method for UT robotic inspection is to utilize UT probes as an “in-process” path planner and NDE inspection device. One work has been done operating a UT controlled robot, which the UT probe dictates robot path planning via real-time processing. This system can determine the trajectory of the UT probe for surface mapping from a single probe start position alone, while also obtaining volumetric evaluation of a curved steel sample^28^.

## Proposed framework for robotic NDE 4.0 surface reconstruction

A new framework for conducting autonomous scanning on complex parts from surface scanning NDE techniques is proposed. The first process of this framework starts with the initial environment reconstruction. This is followed by localizing the robot by registration of the environment to the scanning workspace. The next step is toolpath generation, in which a novel method is proposed: ray-triangle intersection arrays.

This process involves projecting rays onto the initial mesh and checking for intersections, resulting in a zig-zag pattern of “waypoints” holding position and tilt information for the probe on the toolpath. This provides robust scan profiles for any input mesh, albeit with smoothing limitations with respect to tool movements compared to b-splines. Figure 1 illustrates the considered framework depicting the reconstruction-to-NDE scanning process. Following path computation, the generated path is transmitted to the robot, initiating the NDE scanning process. The NDE scan synchronizes the recorded position of the robot’s end effector with the NDE data, generating NDE point data within 3D space respective to the robot’s workspace. This resultant data undergoes post-processing, involving actions such as removing trend effects or converting the 3D NDE point data into a mesh.

### Geometric point cloud from physical environment

The goal of this section is to define an output to a cyber environment $E_{cy}$ based on a physical environment that will be used for path planning. Starting with the basics, the raw output from a reconstruction device is a point cloud labeled as a set of points P. This process is known as point cloud reconstruction. Eventually, P needs conversion into a mesh, forming the reconstructed environment $E_{cy}$. A point cloud P contains a local set of points with indices $p_i$, holding vertex information $t_{xyz}$ and color information *rgb*. Therefore, $p_i = [t_{xyz_i}, rgb_i]$, and $P = [p_1, p_2, p_3, \ldots , p_{LP}]$, where LP is the number or “length” of points in the point cloud. Point clouds are obtained by an “observer,” which in this case would be the reconstruction device of choice.

**Figure 2:**
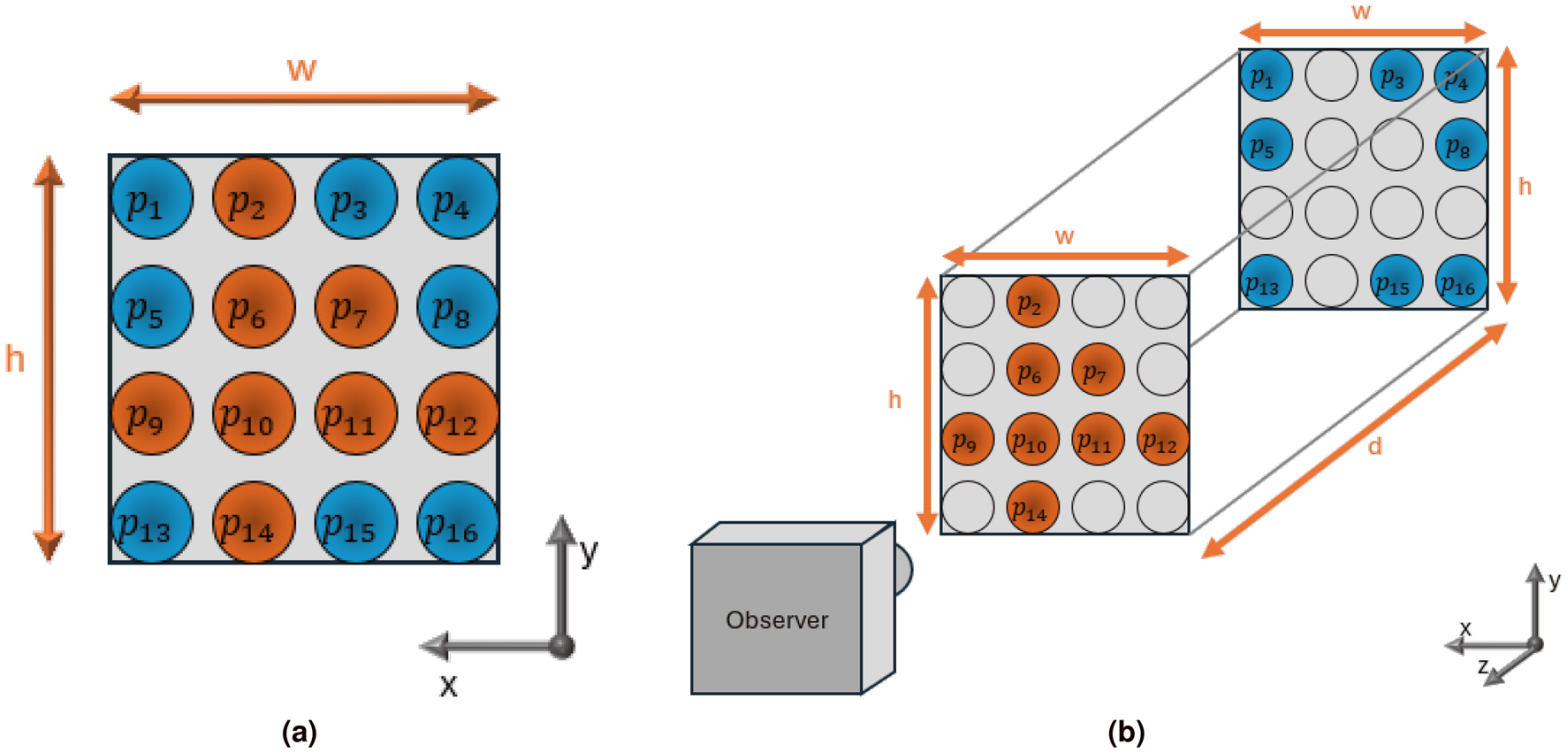
Point cloud example from different perspectives, with a $4\times 4$ grid-like point cloud with area $w\times h$ two sheets of points at different depths defined by d. (**a**) Bird’s eye perspective. (**b**) Isometric perspective.

From point clouds obtained from stereo cameras, the points are typically organized in a grid-like fashion along local coordinates x and y, with variations along z to represent depth. However, the output is unraveled into a 1D vector form. For example, if a stereo camera has a resolution of 1920$\times$1080 points, then LP = 2073600 in the 1D vector $P_{cy}$. Each point falls within width w and height h boundaries local to the point cloud’s frame, creating a grid-like formation of points. Parameters w and h depend on the camera’s field of view and the distance between a physical component inside the workspace. An example is shown in Fig. 2. In this example, a 4 $\times$ 4 grid-like point-cloud is shown, with dimensions $w \times h \times d$ for width, height, and depth. From the point cloud definition, $P_{cy} = [p_1, p_2, p_3 \ldots , p_{16}]$ with LP = 16 and the orange points are closer to the observer than the blue points. The clear points are just placeholders to show orthogonality between the depths, as in, the arrays will hold the same x and y positions with depth. From a reconstruction device, the depth of these points should move along the surface geometry of the environment to reconstruct along the grid. Points that are not visible to the device are defined as p = null and are not considered in the output reconstructed point cloud $P_{cy}$.

### Mesh generation from point cloud

From the point cloud generated, a mesh is generated by connecting vertices together. This process is known as mesh reconstruction. This will obtain a list of faces that will define the surface profile of the physical environment within virtual space. Covered are the basic principles of faces generated from reconstruction and its relation to path planning, with a breakdown shown in Fig. 4. Two popular algorithms are Poisson reconstruction^29,30^ and Delaunay triangulation^31^.

**Figure 3:**
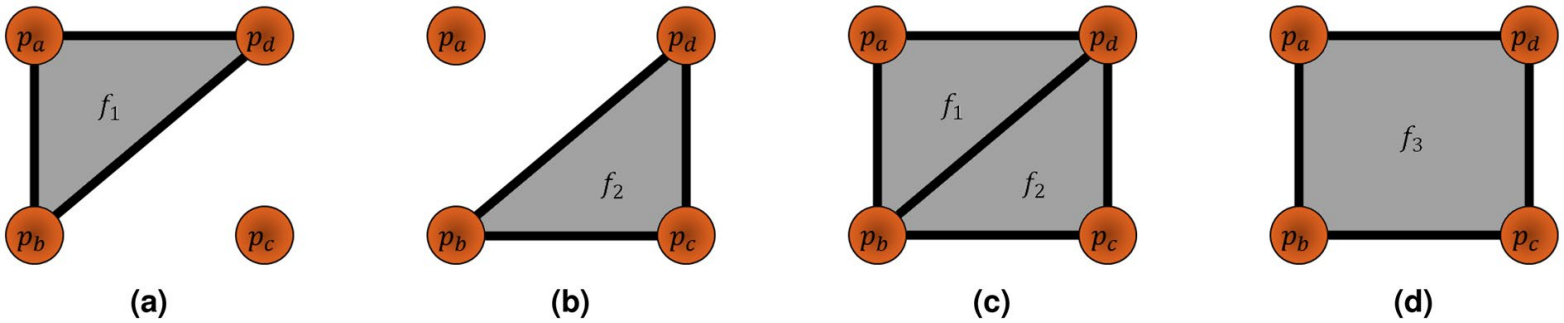
Example showing face formations from vertices, oriented to the reader with $LP=4$. The properties of each formation is shown in Table 1.

A mesh comprises a set of faces F with face indices fi, where $F = [f_1, f_2, \ldots , f_{LF}]$, and LF is the number of faces. Faces are constructed from vertices, with $f_i = [p_1, p_2, \cdots , p_{LP_F}]$, where $LP_F$ is the number of vertices in a face with $LP_F \ge 3$ and $LP_F \ge LF+2$ assuming $LF \ge 1$. Vertices function as the foundation of meshes and are defined from P through reconstruction. An example of faces defined by vertices is shown in Fig. 3. The vertex list V differs subtly from P since reconstruction may reorganize V. Mesh processing may further reorganize V. Although the organization of faces through vertex indexing is abstracted, it is mentioned for contextual purposes. Output data sets typically use this organization to significantly reduce the redundancy of overstated vertices.

**Table 1:** Properties of different face formations from a set of vertices, shown in Fig. 3.

Formation	$F = [\cdots ]$	*LF*
(a)		
a	$f_1$	1
b	$f_2$	1
c	$f_1, f_2$	2
d	$f_3$	1

Face	Points contained	$LP_F$
(b)		
$f_1$	$[p_a, p_b, p_d]$	3
$f_2$	$[p_b, p_c, p_d]$	3
$f_3$	$[p_a, p_b, p_c, p_d]$	4

Table (a) shows the configuration for each set of faces *F*, while table (b) shows the formation of each point per face, which direction of points is relevant for normal calculations per face on the mesh.

Faces are essential in the ray-triangle intersection array algorithm later discussed. Faces contain a flat area between its vertices. A point of intersection will lay on this area, determining probe positioning. Faces also contain a single normal throughout their area, determining probe tilt. A normal from a triangular face, or $LP_F= 3$, may be calculated from the following equation: $n_f = \frac{c}{||c||}$, with $c = v_a v_b \times v_a v_c$ for a face $f = [v_a, v_b, v_c]$. Faces may be triangulated using face culling algorithms^32^.

The order of vertices defined by a face is important for normal orientation. The position of vertices per face determines facial orientation, including the normal or the direction the face is pointing towards. For example, $f=[v_a,v_b,v_c]$ in a counterclockwise formation will face the normal in a positive orientation, while $f=[v_a, v_c, v_b]$ in a clockwise formation will flip the normal in the negative direction. In other words, an incorrect vertex order will flip the probe in the opposite direction, inevitably leading to a collision between the probe and sample!

**Figure 4:**
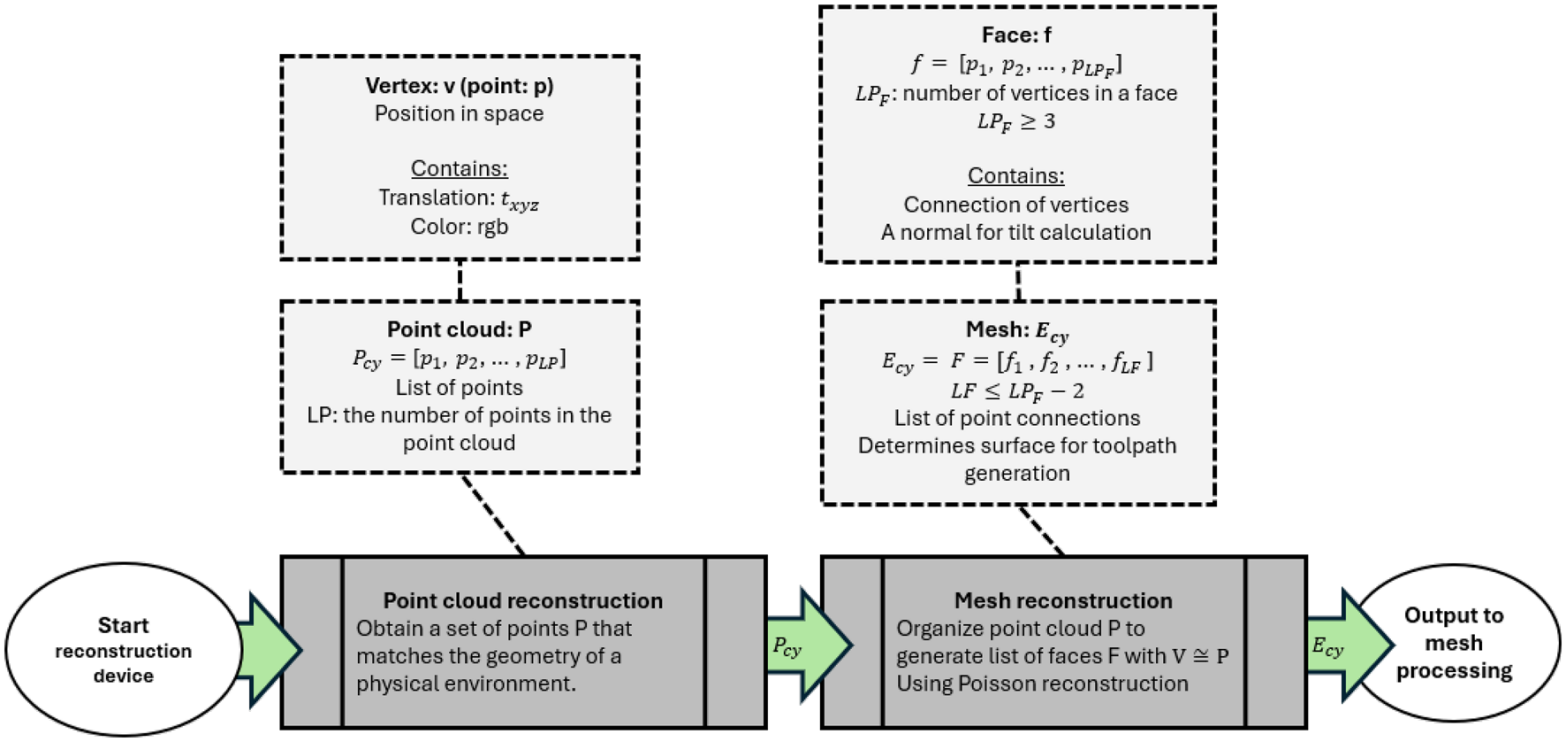
Breakdown of process of mesh reconstruction and properties. The process involves obtaining a point cloud $P_{cy}$ from a reconstruction device, then obtaining surface information through mesh reconstruction. The results are placed for further processing.

### Scanning environment post-processing

Post-processing on the mesh is recommended for effective path planning. Cropping out background components is recommended to prevent manipulation in areas outside the scanning region. This step may also aid in eliminating curved beveling on edges of a sample that merges with background components, a common occurrence during reconstruction. Additionally, smoothing the scan surface is recommended to avoid issues with coarse surfaces that might lead to overcompensation in the rotation of the actuation system. While this article does not delve into the optimization and automation of post-processing procedures, techniques such as manual face cropping, face simplification or decimation, and Laplacian smoothing^33^ are commonly employed to enhance path planning and scanning results.

### Background removal

In a workspace, two classifications of physical objects are considered within a point cloud snapshot: the sample to reconstruct and the background. The toolpath generation algorithm aims to retain only the sample under test, excluding other components. It is crucial to identify what qualifies as background in the workspace. This may include holders like vices, which may appear attached to the sample and need removal. If the sample is on a table, the table itself is considered part of the background. Components outside the robot’s workspace might also appear in the snapshot as background, including the robot and any related wires to the NDE probe. It is essential to note that background components may be important for collision detection. Thus, it is recommended to keep one mesh with the necessary background for collision detection and another for path planning.

To eliminate backgrounds, two methods were employed: manual removal and statistical outlier removal. Statistical outlier removal was a default technique applied to stray point clouds to prevent them from affecting the mesh reconstruction process. Manual cropping was also employed, typically if a background component, such as vices or tables, merged with the sample. For future works, an autonomous solution for background removal will be considered.

## Path Planning: Ray-triangle intersection array algorithm

**Figure 5:**
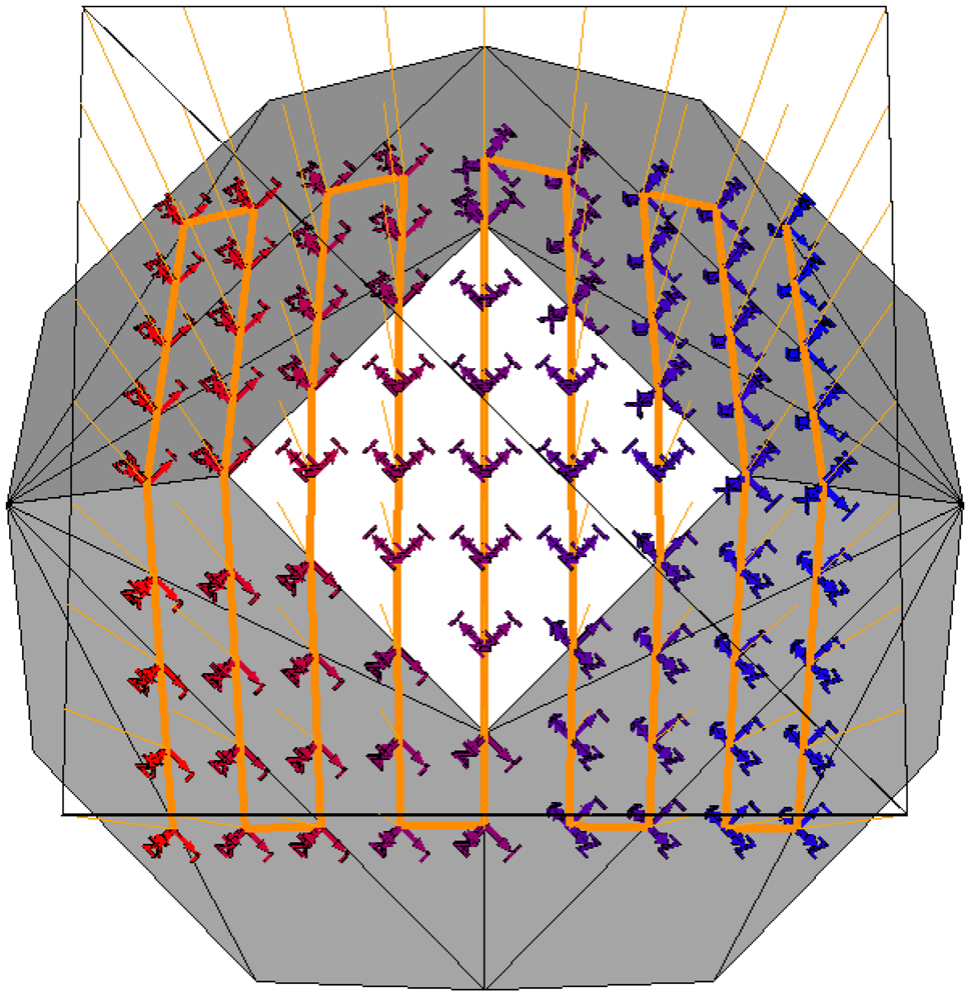
Path planning example using a custom 3D engine, showing the grid of arrays intersecting with a low polygon sphere. Each waypoint is seen, organized in a zig-zag pattern starting from red to blue, where position and rotation is demonstrated. Per each face from respectively combined triangles, the rotation remains constant, but changes for every other face to match the normal project of associated triangles.

The path planning process is expected to produce a linear path of waypoints, W, derived from the processed reconstructed environment mesh, $E_{cy}$. Each waypoint w within W includes translation and rotation properties. W serves as input for the inverse kinematics algorithm, generating the joint set as the toolpath where the NDE probe actuates.

A novel approach was employed to derive W from $E_{cy}$, using ray-triangle intersection arrays. This necessitated the development of a customized 3D engine capable of calculating ray-to-triangle intersections along an input mesh. $E_{cy}$ requires processing to eliminate obstructive environmental components and smooth the surface.

A ray is simply two vertices forming a line in space. Ray-triangle intersection, often implemented through the Möller Trumbore algorithm^34^, allows a ray to intersect with a triangular face. This intersection will provide the location of the waypoint, and the normal of the intersected face for the rotation of the waypoint. Euler rotations can be derived from $n_f$. To ensure the correct direction, the normal direction is flipped to align the probe with the surface. Otherwise, the probe will have a flipped orientation inside the sample which will inevitably cause collision and improper scanning. If a ray does not intersect with the mesh, then it is ignored. Figure 5 shows a zig-zag patern on a low-polygon sphere with intersections and approximate rotations per face.

The objective is to create a grid of rays, each checking the mesh for position and rotation properties of a waypoint. This grid may be envisioned as a formation of rays in a rectangular prism, oriented on the spot to examine on the mesh. The volume of the prism is defined by the scan zone area and the ray length. The ray length should be defined finitely, though may be technically infinite or a high number without affecting the algorithm. The number of rays per row and column represents the resolution of the toolpath. For simple raster scans, rows indicate the number of “swipes” the NDE sensor will move along, and columns represent the number of movements per swipe. Larger numbers of columns enhance fidelity over curved surfaces, though in practice, the robot may “stutter” at each of these points, increasing scan time due to deacceleration and reacceleration.

Once the waypoint path, W, is determined, the joints to actuate the NDE probe must be resolved through inverse kinematics. This process is executed by inputting a finite transformation of the probe, and the joint solution is output. This can be done through simulation, then submitted to the physical robot. As inverse kinematics presents an infinite number of joint configurations, there may be a resolution for an optimal set of joint movements to optimize scan times. This can be done by reducing the movement time of the joints. Inverse kinematics needs to also avoid singularities or impossible movements. Inverse kinematics is solved with RoboDK, a commercial software tremendously helpful for robot arm integration.

It is advantageous to organize points to optimize scan times, often using zig-zag raster scanning. For example, because rays may not intersect, a simple raster algorithm may increase scan time or obtain unwanted data. For the implementation of this algorithm, including zig-zag and line-by-line instances and optimization using a greedy traveling salesman problem (TSP) approach, refer to^35^. TSP approaches create more advanced paths to increase scan speeds at the cost of computation time. More advanced algorithms may also incorporate collision avoidance with background and sample, if the sample contains geometries that may cause self-collision.

## Results and validation of the proposed framework for complex surface reconstruction

Experiments were conducted that demonstrated reconstruction relevant for later path planning. The first shown are the capabilities of reconstruction devices for path planning, and the second are show the capabilities of NDE scanning. Stereovision and Structured light methods were used to obtain a cyber environment from the sample under test. Initially, an Intel RealSense D425i camera used for stereovision was considered. This camera has a depth accuracy of $2\%$ or 40 mm at 2 m lift-off, and output field-of-vision (FOV) of $87^\circ \times 58^\circ$, and an ideal range for acquisition between 0.3 to 3 m. However, as shown later, it had poorer performance than the Creality CR-Scan 01 structured light sensor. This sensor has a depth accuracy at 0.1*mm*, a reconstruction area $53.6\times 37.8 \textrm{cm}^2$ for FOV, and a required lift-off range of 1 m.

**Figure 6:**
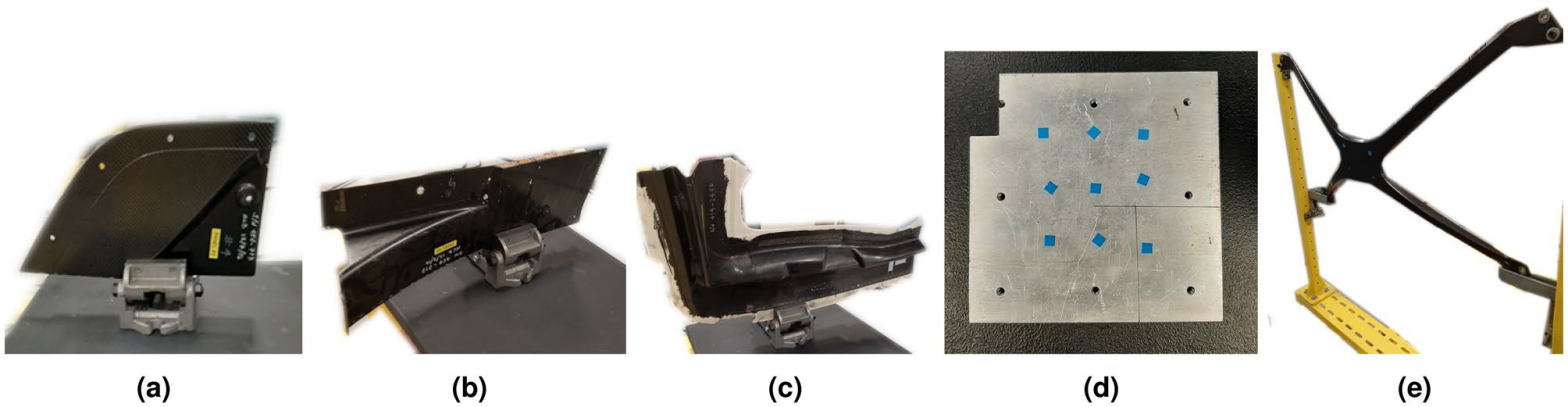
Samples for testing and their respective labels.

Five samples were selected with various geometries, shown in Fig. 6 and general dimensions shown in Table 2. Car samples S1 through S3 have variances in thickness, due to regions having honey-comb structures and other regions only containing the CFRP surface. These samples have hills and valleys that need to be reconstructed as well.

**Table 2:** Properties of each sample to reconstruct.

Sample	X (mm)	Y (mm)	Z (mm)	Material
1	355.6	228.6	19.05	Carbon-fiber
2	533.4	177.8	76.20	Carbon-fiber
3	711.2	431.8	76.2	Carbon-fiber
4	150.0	150.0	6.0	Aluminum
5	210.0	140.0	20.0	Carbon-fiber

### Mesh processing

The same post processing process was used for each sample: crop out the background, decimate mesh to 1000 faces, and Laplacian smoothing with 10 iterations. Alignment procedures are not covered in this article, though it should be noted that point-pairs picking from CloudCompare was used for registration of meshes back to the robot’s base frame. For environment post processing, CloudCompare for statistical outlier filtering and Poisson reconstruction, and MeshLab for decimation and Laplacian smoothing. Note that CloudCompare and MeshLab are free open-source software that are well built for point cloud and mesh processing.

### Results

**Figure 7:**
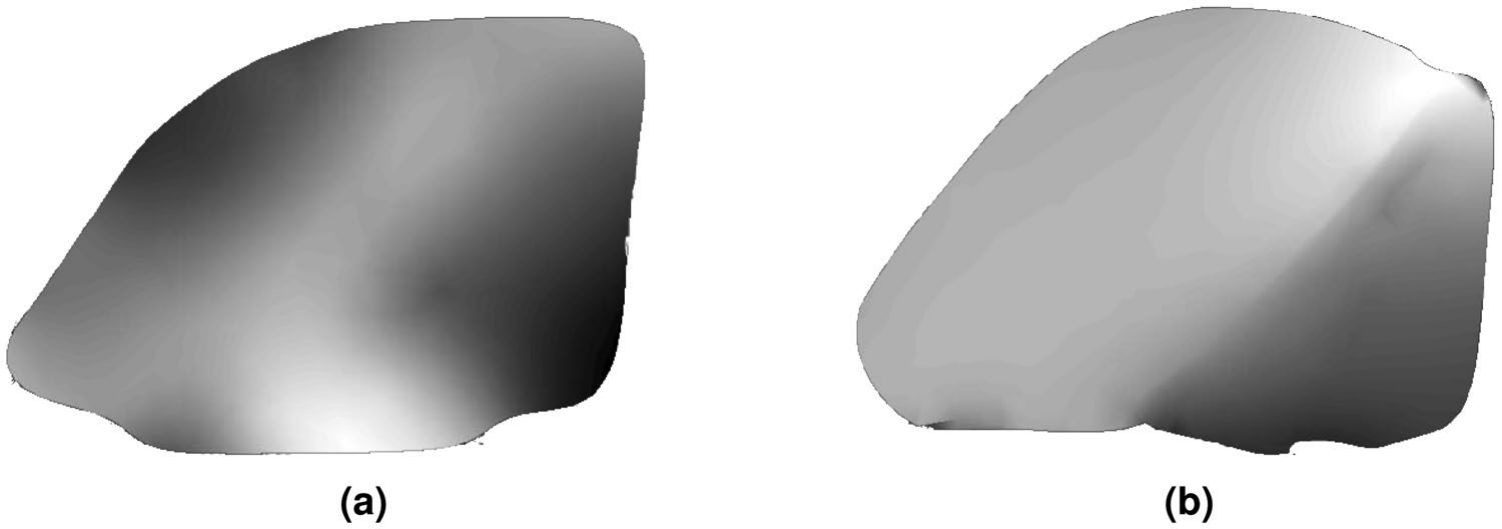
Sample S1 snapshots between the two reconstruction methods. Colormap is set to depth. The following figures will use similar configurations. As seen for the next figures, the stereovision results have a spuratic formation, while the structured light results are more detailed to the physical object. (**a**) Stereovision. (**b**) Structured light.

**Figure 8:**
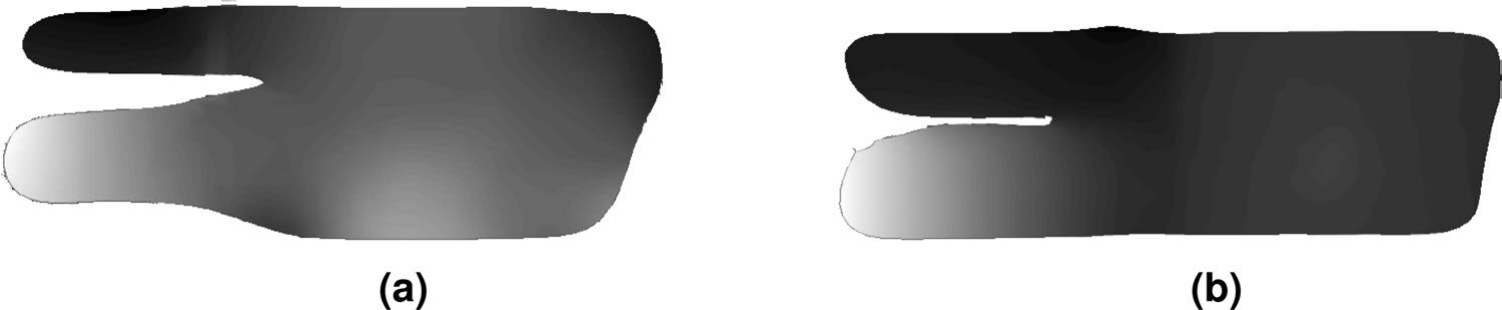
Sample S2 snapshots. (**a**) Stereovision. (**b**) Structured light.

**Figure 9:**
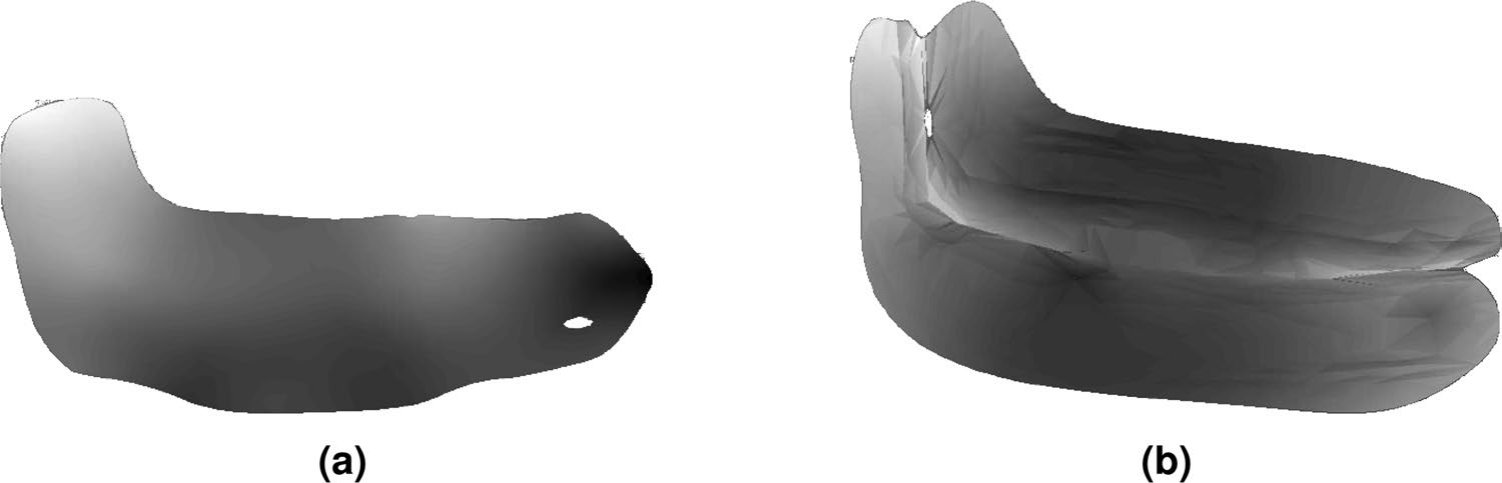
Sample S3 snapshots. (**a**) Stereovision. (**b**) Structured light.

The results for each car piece for samples S1–S3 are shown in Figs. 7, 8, and 9, for both stereovision and structured light. Each colormap is set to depth (white is closer). The first experiment was to use stereovision to reconstruct the three car pieces. Each piece was placed on a vice so that the piece would be orthogonal to the ground while also minimizing any background point cloud data. Otherwise, the background data would merge with the sample, increasing the difficulty of post-processing. The camera was positioned 450*mm* away from each sample. Each was a direct mesh from the Intel Realsense SDK. There are holes that are seen in the snapshots for sample S2 and S3. Specifically, on sample S2, there is a large gap on the left side where the ramp is. The general depths of each are shown, however the resolution is rather poor. Because of this, structured light was considered for further testing. Stereovision may be considered for other applications and its benefits for wider range and portability would be used for NDE 4.0 pathing for unmanned vehicles or drones.

Structured light was used to reconstruct all car samples, the aluminum sample, and the x-brace sample. The position of the camera was dynamic, as the Creality software allows for fast stitching of multiple snapshots while the camera is moving above each sample. This significantly helps increase the size of the area to apply path planning on. It also requires tracking, which has been found difficult to do through the air for certain samples. Because of this, the car samples were placed stationary on a table. Figures 7, 8, and 9 show the results after processing for the first three samples. A glass platform was used to differentiate the sample from the table background. Each sample is shown with higher resolution and depth clarity than the stereo camera. For samples S2 and S3, areas of the mesh are missing, which can be helped by stitching more snapshots together at different viewing perspectives of reconstruction device.

## NDE 4.0 surface scanning on reconstructed specimens using ACUT

**Figure 10:**
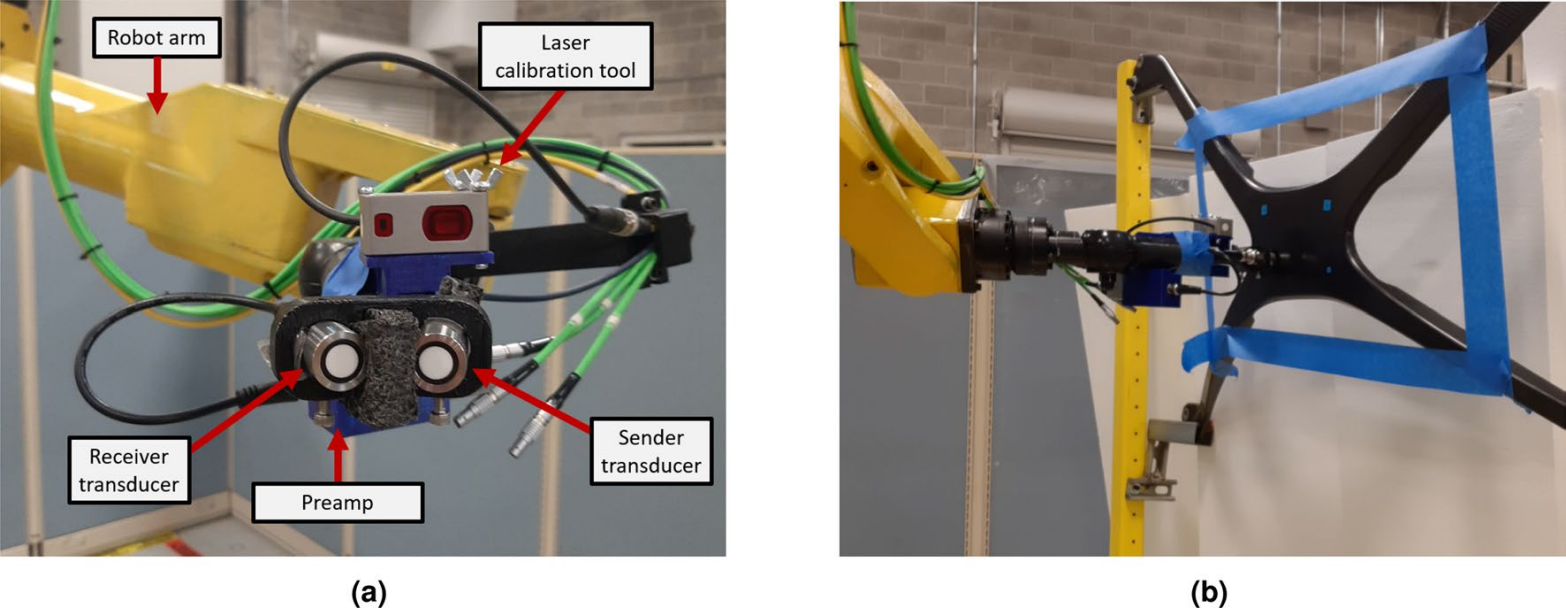
Robot and ACUT probe in physical environment. (**a**) ACUT probe and calibration tool as end-effectors. The tool is held and controlled by the robot arm with its connections shown in Fig. 12. (**b**) ACUT probe conducting a scan on sample S5 (CFRP x-brace). To help reconstruction, foam was placed behind the sample and tape surrounded the area of inspection desired..

**Figure 11:**
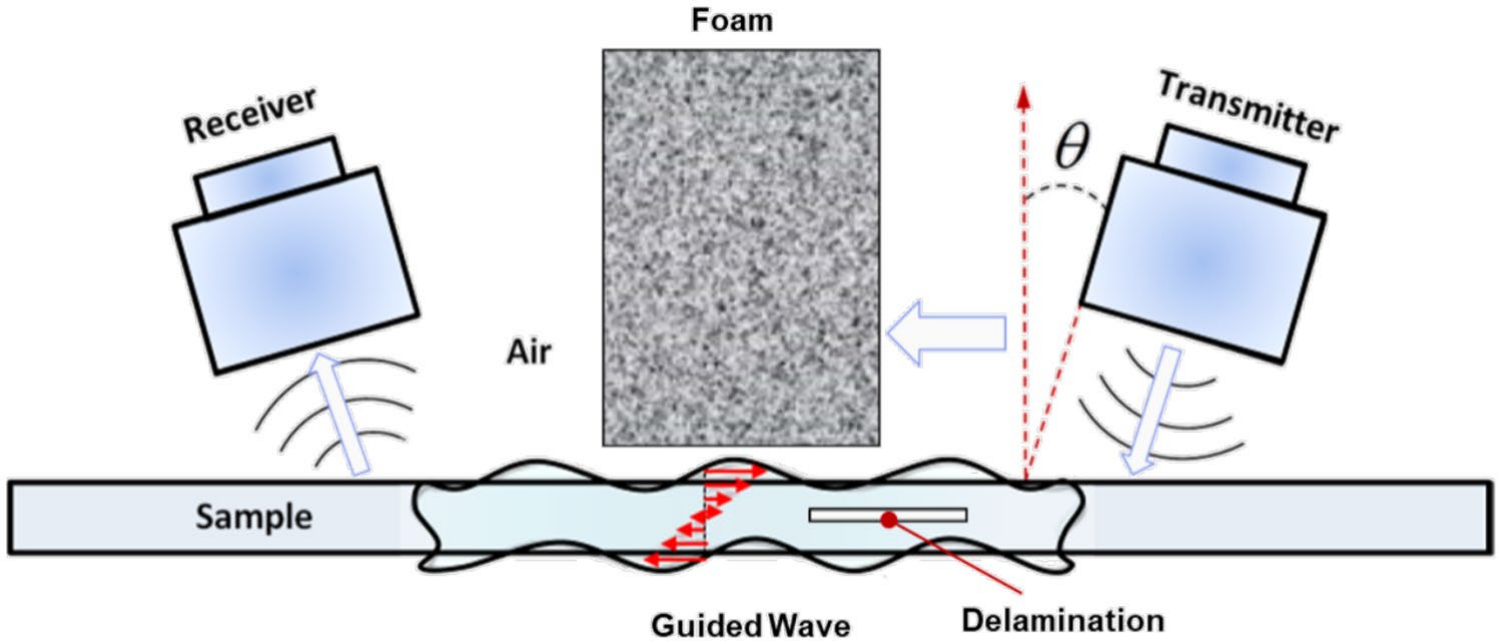
ACUT transducer setup, showing a transmitter and receiver against a sample with an embedded defect. The defect is found through the reflections within the sample, excited from the transmitter which are picked up from the receiver in the air. Foam is placed in between to prevent transmission directly to the receiver.

Once validation was done for using structured light as the main reconstruction method, experiments for UT scanning were next conducted. Anomalies were found using the first order reflection of the air-coupled UT (ACUT) transducers set in pitch-catch mode, with one transmitter sending ultrasonic guided waves and one receiver placed at an angle between each other. Figure 11 illustrates the setup for air-coupled inspection of CFRP test samples using Sonoscan CF200 probes from Sonotec. A SonoAir system was used to excite the probes with a 4-cycle square pulse at 200*kHz* bursts of four at 200*V* and 70*dB* preamp gain. A band pass signal was placed onto the time signal between 180 and 220 *kHz*. To prevent direct wave propagation through the air between the probes, scattering foam is employed. Beneath the foam, waves penetrate the test sample, emerging on the opposite side where they are detected by the receiver. The angle of wave incidence is carefully chosen to activate the fundamental A0 mode within the specimen. The received signal is then amplified using a preamplifier positioned on the robot’s end effector. To streamline the process, measured ultrasonic (UT) signals are automatically time windowed. The energy of the received signal serves as a feature for flaw detection. Figure 10 shows the ACUT sensors and laser calibration tool. A Banner LM150KUQP depth laser was attached to the end effector of the robot to obtain calibration information which ties known physical spots to virtual space. This is done through point-pairs picking in CloudCompare. A Fanuc ARCMate 100iB robot arm capable of 6-DOF movements was used to manipulate the ACUT probes and laser calibration tools.

**Figure 12:**
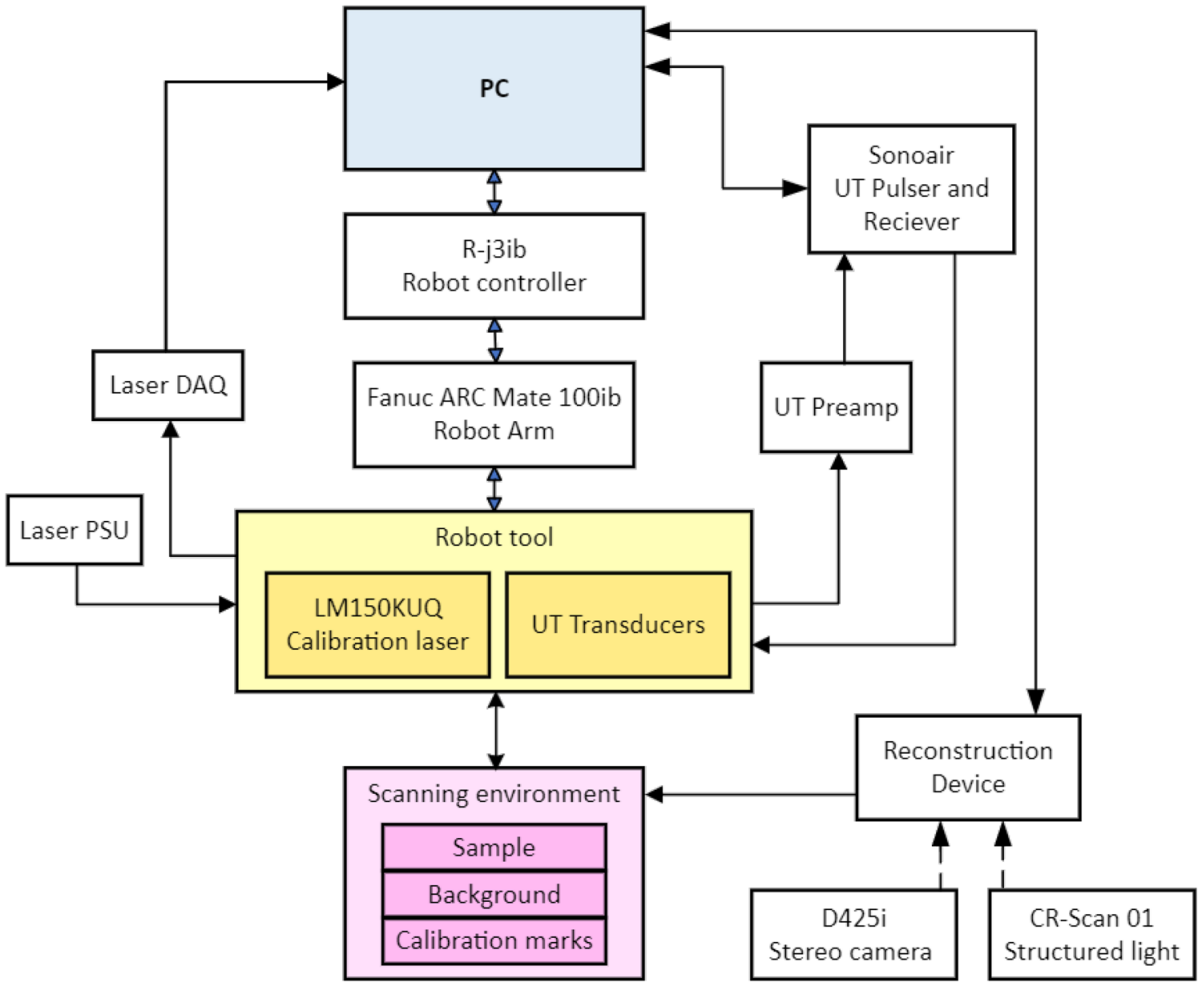
Setup diagram. The robot is controlled by operations sent from the robot controller and PC. The robot holds a tool containing the UT probes and a laser for mesh registration purposes, both connected to the PC for synchronization with the robot’s tool frame. The UT probes will also be excited from a SonoAir system. The sensors on the robot tool, along with the reconstruction device, interact with the scanning environment.

The reconstructed environments were obtained by the Creality CR-Scan 01 structured light sensor. After mesh processing, like the car pieces from samples S1–S3, the meshes were placed into the ray-triangle path generator. The generated toolpath on the mesh is input into RoboDK to create a set of instructions for simulated and physical robots to manipulate the ACUT probe. This simulation sets the ACUT probes lift off to 5 cm. The instructions are then run on the physical robot, in which the robot’s tool orientation is synchronized with the UT signal within the scan duration (Fig. 12).

**Figure 13:**
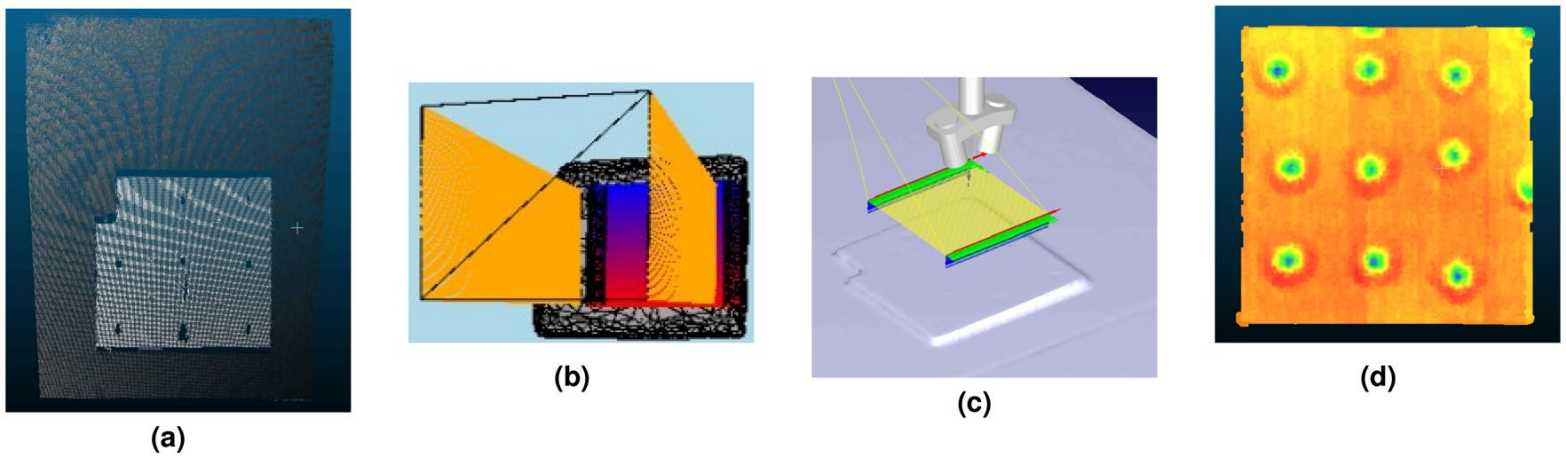
Aluminum sample reconstruction to ACUT results. (**a**) Point cloud from structured light. (**b**) Toolpath generation after mesh processing, with an area of 100 mm $\times$ 100 mm and resolution of 100 $\times$ 2 waypoints. (**c**) Simulated movements in RDK with a global lift-off of 50 mm along the z-axis. (**d**) ACUT surface results, showing surface profile including the calibration stickers.

Samples S4 and S5 shown in Fig. 6 and details in Fig. 2 were used. A flat aluminum block as sample S4 was used as a base reference scan for a simple geometry. This sample includes milled holes and surface calibration stickers for detection. The ACUT surface detection scan and its process are shown in Fig. 13. For the aluminum sample, the nine calibration stickers are clearly visible on this sample. Each sticker was $0.25 \, \textrm{in}^2$ in area and approximately 0.07 mm in thickness, however because of the small area size and convolution, the stickers are rounded into a circular formation. The milled holes were cropped from these results, but they are visible on the top and right corners. There also is a slight gradient indicating that the bottom left of the sample is slightly closer to the robot’s base than the top right, which is a concern for alignment. Despite this, the surface features are clearly picked up.

Sample S5, which will be referred to as an x-brace, contains a complex geometry and is more difficult to detect defects. This sample is 7.5 mm in thickness and contains three subsurface defects with various intensities, with the largest on the bottom left and smallest on the top right. As these scans were taken at separate times, the x-brace was reconstructed twice. To help with this new reconstruction, tape was placed to be used as a reference for tracking using structured light. Two different orientations were evaluated with similar scanning procedures, with the only major differences being the sample’s physical location.

**Figure 14:**
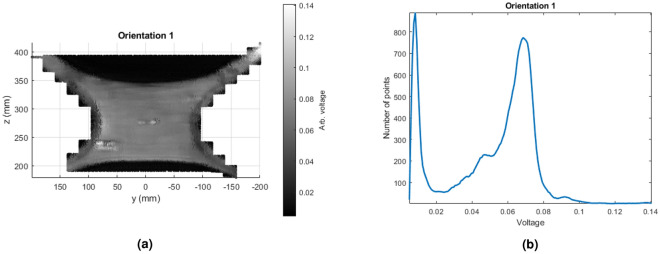
UT subsurface scan air removal, with the air removed results shown next in Fig. 15 for scan 1. The same method was used for scan 2. (**a**) Raw ACUT point cloud containing data within ai, which is notified as black as there is no reflection returned. (**b**) Histogram of raw data used for filtering, showing a large amount of air signal for lower voltages, as well as the peak of voltages indicating the sample background.

**Figure 15:**
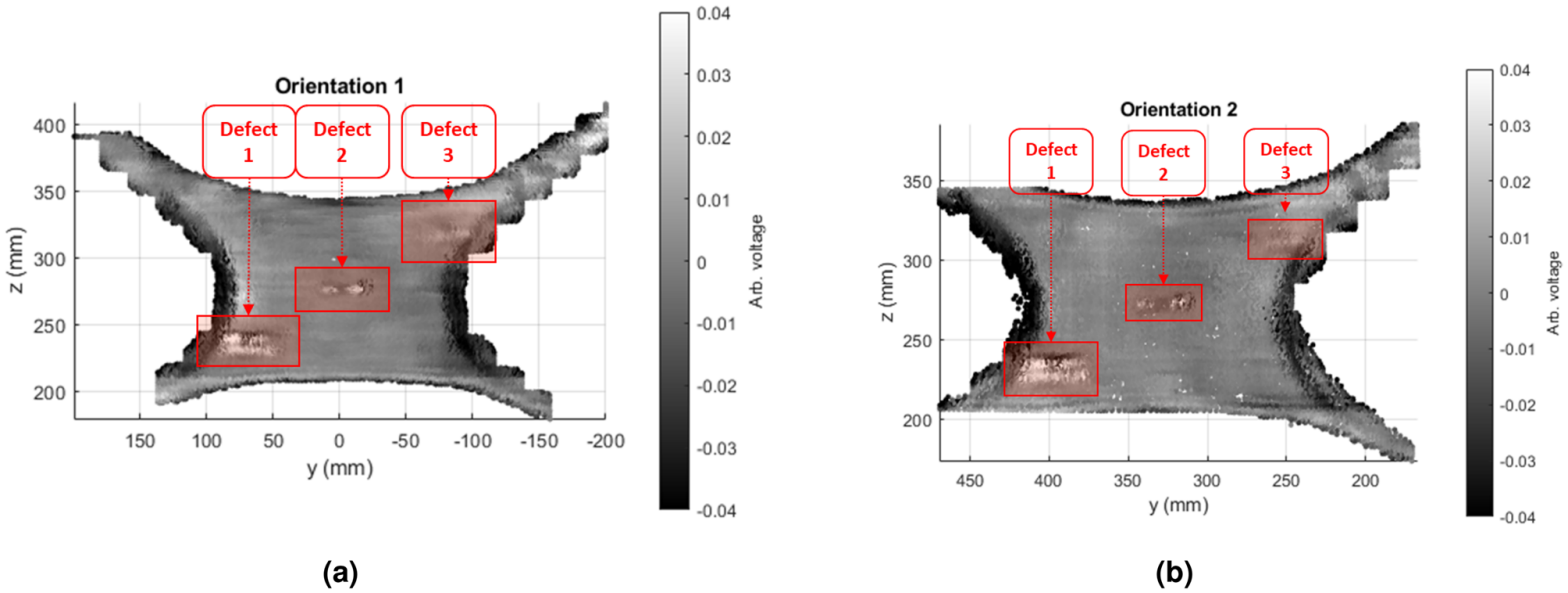
Post-processed ACUT results for both orientations. All three defects are seen in both images, regardless of orientation. (**a**) Scan 1. (**b**) Scan 1.

Post processing involves processing each 3D point cloud using known information about the raster pattern of the scan. The process is shown in Fig. 14 and outputs at Fig. 15. From even the raw data, each defect is visible, albeit the smallest defect found top left is difficult to see. First, signals recorded in the air are removed. This is a straightforward process, as signals in the air will return a lower reflected signal than in the sample. Values under 0.02*V* are removed from the scan. The next step is to apply detrending to alleviate probe orientation errors. This is done throw median subtraction per scan line. As each line is a set of 3D vertices unorganized in space, these needed to be calculated using the initial scan parameters to parse and index each line. Once each scan line is found, simply the median of that line is subtracted. The results for both scans are shown in Fig. 15. Note that after median filtering, then the voltage will be centered around zero rather, hence why further voltage values are considered as arbitrary.

After processing, the defects may be seen around $\pm 0.03$ for the large defect 1, $\pm 0.01$ for the middle defect 2, and $\pm 0.005$ for the smaller defect 3, with the background being close to 0 through the median subtraction detrend, all values in arbitrary voltage units. The edges of the sample also appear dark as well. This is due to the structure of the x-brace having curved edges that may affect the reflected signal, despite attempting to scan along these regions at the proper orientation. The defects also are parallel with the probe’s orientation. For example, if the probe’s local z-axis were rotated $90^\circ$ , the defects would also rotate $90^\circ$ .

## Conclusions and future work

Through the experimental results, it is shown that using structured light environmental reconstruction along with point-pairs picking on calibrated regions for registration is an effective technique to parse surface and subsurface defects with air-coupled UT probes. Importantly, the path planning through the ray-triangle intersection algorithm enables toolpath generation by parsing a position through intersection on the mesh, and rotation through the face from intersection. In turn this also enables autonomy for scanning with only basic parameters such as area, resolution, and location of the inspection area needed to be known. This assumes the processes in the framework, which are environment reconstruction and alignment, are also automated. Reconstruction may be automated by either placing the reconstruction device on the robot arm for mesh reconstruction scanning or placing the device statically beside the area of interest. Registration may be automated by various means, such as April tags or iterative closest point algorithms^36,37^.

As seen in Figs. 13 and 15, the areas of interest are seen versus the sample’s background, either as surface stickers or subsurface defects. Such areas of interest may be found by the robotic system mostly independent of orientation and be localized with respect to the robot for further analysis. The importance of the robot’s awareness of each defect may be for secondary operations after the scan, more-so than just giving acknowledgment for a human operator. For example, if the robot were to have an operation to repair by composite scarfing, then this gives insight on where the operations should take place^38^. Examples exist for this repair on robotic arm systems^39^ and mobile systems^40^. For these secondary operations to be applicable, more information such as the precise depth and the amount of scarfing required would be up to a future endeavor.

There may be more room for different environment sensing research. For the examined stereovision results, it is shown less effective reconstruction than structured light results, which provides significant issues in determining the option for path planning. These issues do not disqualify the practicality of stereovision for more portable systems, such as unmanned vehicles or drones, and these results are down to the sensors used in the experiments. As mentioned previously, there has been research done with stereovision with robotics^23–25^.

The system itself may incorporate more autonomous procedures or otherwise improve robotic accuracy. For example, autonomous movement of the structured light sensor during environment reconstruction may be implemented, by simply conducting a raster-scan above the surface. There have been two issues with this procedure. One is the difficulty parsing an orientation proper to the robot end-effector, which seems to change with an error up to 2*cm*. If using autonomous registration methods such as center-point transformation, this error is not acceptable. Therefore point-pairs picking was used, which used human interaction. This human interaction contradicts the autonomous nature of the setup. The second issue is tracking to expand the workspace of the digital reconstruction by parsing similar point clouds together. This is a built-in feature with Creality structured light software. However, the tracking may be lost during scanning, which halts the expansion of the environmental workspace. A system should be set up that prevents this tracking loss; however, this may require a feedback system between the robot scanning system and the structured light system. Methods to remove merging background components on the mesh, currently done as manual cropping, should also be automated.

For the path planner, there is work that needs to be done. When creating a surface scan, an important feature is the implementation of “jumps”, where a sensor will reposition itself above the sample to prevent collisions. There is also a global lift-off feature, meaning lift-offs will only displace the entire toolpath by a certain amount along only one direction. For cylindrical surfaces such as pipes, this will not work for faces orthogonal to the lift-off direction as the sensor will be placed into the air. To fix this, lift-offs should be based on the normal of the face instead. When applying Poisson reconstruction, the edges of the sample tend to bevel, creating an unwanted curvature for flat samples. Finally, simplification and automation of scanning configuration, such as the orientation of the rectangular prism shooting the array of rays for intersection, should be implemented. There may also be examinations upon the inevitable inaccuracies of robotic arm systems. There has been work in discovering robotic properties that would add to positioning error of probes using x-ray computed tomography^41^.

Future works will examine methods such as eddy current testing using coil arrays using low-weight flex probes on corroded steel specimens. Eddy current array flex probes potentially allow for highly accurate measurements from the probe, gliding along the surface of the sample while obtaining sub-millimeter sized defects at high frequencies and resolutions. For eddy current probes, there are much higher requirements of accurate probe placement, which moving directly on the surface would offset due to constant and low lift-offs. The issue is ensuring the probe is always on the sample without damaging the probe, tool holding the probe, or sample. The path planning procedure would need to compensate for complex movements required for scanning to glide the probe while accurately obtaining the orientation of each coil with respect to the robot. A simplification of the problem, currently under development, is to start is to use an eddy current array in a solid body configuration at a higher lift-off to ensure the coils are within known orientations and the same path planning procedure at the UT may be used, albeit with stricter orientation requirements. There are other considerations such as any conductive piece on the tool or robot that may interfere with the eddy current signal, which is planned to use non-conductive material for the tool with a large distance between the robot and coil, or the phenomena that may occur due to unknown alignment of the probe against the normal of the material’s surface.

Despite shortcomings in terms of path planning and automation, the procedure of reconstruction still holds strong for the robotic arm environment. This can be seen with the surprisingly good UT subsurface results, in which all defects are visible. With more implementation, this system may be an incredible tool for scanning any complex environment regardless of initial orientation within the robot’s workspace. It is hoped that this article’s procedure may be extended to unmanned vehicles and drones.

## Data Availability

The datasets used and/or analysed during the current study available from the corresponding author on reasonable request.

## References

[1] Rani, P. & Agrawal, A. K. Failure analysis of a low-pressure stage steam turbine blade. *Nondestruct. Test. Eval.* 1–15 (2022).

[2] Dorji U, Ghomashch R (2014). Hydro turbine failure mechanisms: An overview. Eng. Fail. Anal..

[3] Singh S, Kharub M, Singh J, Singh J, Jangid V (2021). Brief survey on mechanical failure and preventive mechanism of turbine blades. Mater. Today Proc..

[4] Zimmermann N, Wang PH (2020). A review of failure modes and fracture analysis of aircraft composite materials. Eng. Fail. Anal..

[5] Czaban M (2019). Aircraft corrosion—Review of corrosion processes and its effects in selected cases. Fatigue Aircraft Struct..

[6] Shi, X. *et al.* Ai-enabled robotic NDE for structural damage assessment and repair. *Mater. Eval.* **79**(7), (2021).

[7] Genkuan, L. Analysis of causes of boiler accidents in power plant and accident handling based on mathematical statistics. In *2018 International Conference on Engineering Simulation and Intelligent Control. *17–20 (2019).

[8] Bogue R (2010). The role of robotics in non-destructive testing. Indus. Robot. Int. J..

[9] Mukherjee S (2022). Inline pipeline inspection using hybrid deep learning aided endoscopic laser profiling. J. Nondestruct. Eval..

[10] Xu Z, Liu Y (2019). Abb robotic arm offline programming system. J. Phys. Conf. Ser. (IOP Publication).

[11] Li, Z. & Deng, Y. Quantifying predictive uncertainty in damage classification for nondestructive evaluation using Bayesian approximation and deep learning. *Inverse Probl.* **40**(4), (2024).

[12] Mukherjee, S., Peng, L., Udpa, L. & Deng, Y. Dynamic defect detection in fast, robust nde methods by transfer learning based optimally binned hypothesis tests. *Res. Nondestruct. Eval.* **35**(2), 70–101 (2024).

[13] Lu, Z., Xu, C., Pan, Q., Zhao, X. & Li, X. Inverse kinematic analysis and evaluation of a robot for nondestructive testing application. *J. Robot.* **2015**, 5–5 (2015).

[14] Guo C, Xu C, Hao J, Xiao D, Yang W (2019). Ultrasonic non-destructive testing system of semi-enclosed workpiece with dual-robot testing system. Sensors.

[15] Gripp, S. A twin robot approach for ut inspection and porosity evaluation of complex shaped helicopter components. In *ECNDT 2006* (2006).

[16] Oster, R. Non-destructive testing methodologies on helicopter fiber composite components challenges today and in the future. In *18th World Conference on Nondestructive Testing*. 16–20 (2012).

[17] Lu Z, Xu C, Pan Q, Meng F, Li X (2015). Automatic method for synchronizing workpiece frames in twin-robot nondestructive testing system. Chin. J. Mech. Eng..

[18] Yan, Y. *et al.* Nondestructive testing of composite fibre materials with hyperspectral imaging: Evaluative studies in the EU h2020 FibreEUse project. arXiv preprint arXiv:2111.03443 (2021).

[19] Kang R, Guerrero P, Probst G, Slaets P, Dewulf W (2022). Reference free method for robot CT imaging geometry estimation. Precis. Eng..

[20] Herl G, Hiller J, Maier A (2020). Scanning trajectory optimisation using a quantitative tuybased local quality estimation for robot-based X-ray computed tomography. Nondestruct. Test. Eval..

[21] Khan, A., Mineo, C., Dobie, G., Macleod, C. N. & Pierce, S. G. Introducing adaptive vision-guided robotic non-destructive inspection. *Rev. Prog. Quant. Nondestruct. Eval.* 1–4 (2019).

[22] Alenyà G, Foix S, Torras C (2014). TOF cameras for active vision in robotics. Sens. Actuators A Phys..

[23] Roudari, S. S., Okore-Hanson, T., Hamoush, S., Yi, S. & Megri, A. Robotic nondestructive evaluation of RC structures using 3D vision camera, IE, and GPR. In *American Society for Nondestructive Testing* (2019).

[24] Chen, X.-Z., Huang, Y.-M. & Chen, S.-b. Model analysis and experimental technique on computing accuracy of seam spatial position information based on stereo vision for welding robot. *Indus. Robot Int. J. ***39**(4), 349–356 (2012).

[25] Gilmour, A. *et al.* Robotic positioning for quality assurance of feature-sparse components using a depth-sensing camera. *IEEE Sens. J.* **23**(9), 10032–10040(2023).

[26] Galleguillos C (2015). Thermographic non-destructive inspection of wind turbine blades using unmanned aerial systems. Plast. Rubber Compos..

[27] Mineo C, Montinaro N, Fustaino M, Pantano A, Cerniglia D (2022). Fine alignment of thermographic images for robotic inspection of parts with complex geometries. Sensors.

[28] Mineo C, Cerniglia D, Poole A (2022). Autonomous robotic sensing for simultaneous geometric and volumetric inspection of free-form parts. J. Intell. Robot. Syst..

[29] Michael Kazhdan, M. B. & Hoppe, H. Poisson surface reconstruction. In *Eurographics Symposium on Geometry Processing.* **7**(4), 61–70 (2006).

[30] Kazhdan, M. & Hoppe, H. Screened Poisson surface reconstruction. In *ACM Transactions on Graphics.* **32**(3), 1–13(2013).

[31] Boissonnat, J.-D. & Geiger, B. Three-dimensional reconstruction of complex shapes based on the delaunay triangulation. In *Biomedical Image Processing and Biomedical Visualization*. Vol. 1905. 964–975 (SPIE, 1993).

[32] De Vries, J. *Learn Opengl*. *Licensed Under CC BY*. Vol. 4 (2015).

[33] Sorkine-Hornungl, O. Laplacian surface editing. In *Eurographics Symposium on Geometry Processing*. 175–184 (2004).

[34] Moller, T. & Trumbore, B. Fast, minimum storage ray/triangle intersection. In *ACM SIGGRAPH 2005 Courses*. 7-es (2005).

[35] Hamilton, C. N. *5D Nondestructive Evaluation: Object Reconstruction to Toolpath Generation*. Master’s thesis, Michigan State University (2021).

[36] Zheng, X., Ma, R., Gao, R. & Hao, Q. Phase-slam: Mobile structured light illumination for full body 3D scanning. In *2021 IEEE/RSJ International Conference on Intelligent Robots and Systems (IROS)*. 1617–1624 (IEEE, 2021).

[37] Wang, J. & Olson, E. Apriltag 2: Efficient and robust fiducial detection. In *2016 IEEE/RSJ International Conference on Intelligent Robots and Systems (IROS)*. 4193–4198 (IEEE, 2016).

[38] Breuer, U. P. Commercial Aircraft Composite Technology. **115** (2016).

[39] Hoefener, M. & Schueppstuhl, T. Small industrial robots for on-aircraft repair of composite structures. In *ISR/Robotik 2014; 41st International Symposium on Robotics*. 1–6 (VDE, 2014).

[40] Negri SP (2019). A modular mobile robotic architecture for defects detection and repair in narrow tunnels of CFRP aeronautic components. Robot. Comput.-Integr. Manuf..

[41] Kang R, Probst GM, Slaets P, Dewulf W (2020). Investigation of the impact of various robot properties on a twin robot-CT system. Nondestruct. Test. Eval..

